# Disentangling the symptoms of schizophrenia: Network analysis in acute phase patients and in patients with predominant negative symptoms

**DOI:** 10.1192/j.eurpsy.2021.2241

**Published:** 2021-10-13

**Authors:** Koen Demyttenaere, Nicolas Leenaerts, Károly Acsai, Barbara Sebe, István Laszlovszky, Ágota Barabássy, Laura Fonticoli, Balázs Szatmári, Willie Earley, György Németh, Christoph U. Correll

**Affiliations:** 1 Faculty of Medicine, Department of Neurosciences, Psychiatry Research Group, University of Leuven, Leuven, Belgium; 2 University Psychiatric Center, KU Leuven, Leuven, Belgium; 3 Medical Division, Gedeon Richter Plc., Budapest, Hungary; 4 Medical Affairs Corporate, Recordati, Milan, Italy; 5 AbbVie, Madison, New Jersey, USA; 6 Department of Psychiatry, Northwell Health, The Zucker Hillside Hospital, Glen Oaks, New York, USA; 7 Department of Psychiatry and Molecular Medicine, Donald and Barbara Zucker School of Medicine at Hofstra/Northwell, Hempstead, New York, USA; 8 Department of Child and Adolescent Psychiatry, Charité Universitätsmedizin Berlin, Berlin, Germany

**Keywords:** Cariprazine, negative symptoms, network analysis, Positive and Negative Syndrome Scale, symptom factors

## Abstract

**Background:**

The Positive and Negative Syndrome Scale (PANSS) is widely used in schizophrenia and has been divided into distinct factors (5-factor models) and subfactors. Network analyses are newer in psychiatry and can help to better understand the relationships and interactions between the symptoms of a psychiatric disorder. The aim of this study was threefold: (a) to evaluate connections between schizophrenia symptoms in two populations of patients (patients in the acutely exacerbated phase of schizophrenia and patients with predominant negative symptoms [PNS]), (b) to test whether network analyses support the Mohr 5 factor model of the PANSS and the Kahn 2 factor model of negative symptoms, and finally (c) to identify the most central symptoms in the two populations.

**Methods:**

Using pooled baseline data from four cariprazine clinical trials in patients with acute exacerbation of schizophrenia (*n* = 2193) and the cariprazine–risperidone study in patients with PNS (*n* = 460), separate network analyses were performed. Network structures were estimated for all 30 items of the PANSS.

**Results:**

While negative symptoms in patients with an acute exacerbation of schizophrenia are correlated with other PANSS symptoms, these negative symptoms are not correlated with other PANSS symptoms in patients with PNS. The Mohr factors were partially reflected in the network analyses. The two most central symptoms (largest node strength) were delusions and uncooperativeness in acute phase patients and hostility and delusions in patients with PNS.

**Conclusions:**

This network analysis suggests that symptoms of schizophrenia are differently structured in acute and PNS patients. While in the former, negative symptoms are mainly secondary, in patients with PNS, they are mainly primary. Further, primary negative symptoms are better conceptualized as distinct negative symptom dimensions of the PANSS.

## Introduction

Since the first clinical descriptions of schizophrenia, the presence of both “excess” symptoms, such as delusions and hallucinations, and “deficit” symptoms, such as a reduced intellectual, social, or emotional functioning, has been acknowledged [1, 2]. Around 50 to 60 years ago, de Clérambault and Henri Ey introduced the term positive symptoms in place of “excess” symptoms and the term negative symptoms to relabel “deficit” symptoms [3, 4]. Later, Crow and others proposed that positive and negative symptoms were the basis for two types of schizophrenia [5]. One is an “acute form” with mainly positive symptoms, and the other a “deficit state” characterized by predominant negative symptoms (PNS). This proposal coincided with an increased interest in which symptoms would fall under the category of negative symptoms [6]. This development of the classification of symptoms led to the creation of the Positive and Negative Syndrome Scale (PANSS), which has become a widely used symptom scale in clinical trials of schizophrenia [7]. Its 30 items are subdivided into 7 positive, 7 negative, and 16 general items.

Despite being the gold standard symptom scale for schizophrenia, many attempts have been made to better cluster the individual PANSS items into factor models with 2, 3, 5, 7, 8, and even 11 factors [8–11]. The 5-factor structure, which generally includes positive, negative, cognitive/disorganization, depression/anxiety, and excitability/hostility domains, is the basis of most models, including the Lindenmayer, Marder, Mohr and the latest Wallwork models [9–12]. Although several factor analyses studies have suggested that a 5-factor model captures PANSS structure better than the original PANSS subscales, no single model has achieved broad consensus, and the three original subscales are still widely used [12]. Some inconsistent results have also been noted, which may be due to methodological considerations across the studies (e.g., sampling methods, illness stage during which participants were assessed, and statistical methods) [13].

Ongoing dissatisfaction with the PANSS classification, in particular the classification of negative symptoms, pushed the National Institute of Mental Health to sponsor the 2005 Development Conference on Negative Symptoms [14]. A recommendation was given to develop new rating scales for the assessment of negative symptoms, which led to the development of the Brief Negative Symptom Scale (BNSS) [15] and the Clinical Assessment Interview for Negative Symptoms (CAINS) [16], leading to a consensus on which symptoms are negative symptoms: avolition, anhedonia, alogia, blunted affect, and asociality. Further, regarding negative symptoms, three other points of debate were raised: the differentiation between primary and secondary negative symptoms, their uni- or multidimensionality, and the differentiation between transient and persistent negative symptoms [17–26].

First, avolition, anhedonia, alogia, blunted affect, and asociality can be both primary symptoms (part of schizophrenia) or secondary due to positive symptoms (e.g., withdrawal due to paranoid delusions), depression (e.g., anhedonia, avolition, and asociality), use of antipsychotics (e.g., extrapyramidal side effects and sedation), or substance abuse or environmental deprivation (hospitalization or lack of psychosocial stimuli). As such, it is important to differentiate primary from secondary negative symptoms as they might require different interventions [27]. Second, the one-dimensional structure of negative symptoms has been abandoned in favor of newer models, ranging from 2- to 5-factor models. More recent factor analyses provided evidence for a two-dimensional structure with experiential (avolition, apathy, and lack of energy) and expressive (blunted affect, poverty of speech) deficits [18, 28, 29]. These factors may be associated with different underlying neurobiological abnormalities and psychosocial outcomes [19]. For example, the expressive factor may have a stronger association with nonsocial and neuropsychological function, while the experiential factor is theorized to have a direct impact on functional outcomes in schizophrenia [25]. The concept behind the 2-factor experiential and expressive deficit model for negative symptoms has been integrated into the Diagnostic and Statistical Manual of Mental Disorders Fifth Edition (DSM-5), specified as “diminished emotional expression or avolition” [26]. Other studies have provided support for a 5-factor structure, where anhedonia, asociality, avolition, blunted affect, and alogia are all separate factors [20]. Third, more recently, it has been suggested that the distinction between transitory and persistent negative symptoms could be as important as the distinction between primary and secondary negative symptoms when looking at long-term outcome [22, 23]. Persistent negative symptoms are characterized by: (a) the presence of at least three moderate negative symptoms or two moderately severe negative symptoms, (b) by a defined upper threshold for positive symptoms, depression and extrapyramidal symptoms on accepted and validated rating scales, and (c) most importantly the persistence of negative symptoms of sufficient severity for at least 6 months [27, 30]. In clinical trials, as requested by regulatory agencies, further concepts have been introduced for the assessment of negative symptoms: the “predominant negative symptoms” and “prominent negative symptoms.” Predominant negative symptoms are characterized by moderate-to-severe negative symptoms that have greater relative severity than co-occurring positive symptoms, whereas prominent negative symptoms just refer to the severity of negative symptoms (moderate-to-severe) without any reference to positive symptoms [27, 30].

The ultimate aim of identifying reliable symptom factors is to better subtype patients and match between patient subgroups and treatment options. Recently, network analyses have been introduced in psychiatry to better understand the relationships and interactions between symptoms of a given psychiatric disorder. In such analyses, symptoms are seen as a network or a system of entities that have connections with each other and can influence one another [31, 32]. Network analyses allow for new conceptualizations of mental disorders where symptoms can be ranked according to their centrality (number and strength of connections with other symptoms of the disorder) and where visualization of the relationships enables us to see which symptoms are closely related to each other. For example, a network analysis of BNSS items revealed that the latent structure of negative symptoms consisted of six negative symptom domains (i.e., anhedonia, avolition, asociality, blunted affect, alogia, and lack of normal distress) generally corresponding to the established negative symptom constructs except for “lack of normal distress,” which is a reduction in the intensity or frequency of negative emotional experience [33]. Although this factor was identified in the BNSS studies as a potential sixth negative symptom factor, this symptom did not clearly load neither on the Experiential Domain, nor on the Expressive Deficit and results across studies were inconsistent. Therefore, in a newer consensus paper of the European Psychiatric Association it is suggested for further studies to clarify whether “lack of normal distress” really belongs to the current negative symptom construct or to other psychopathological constructs [30]. In another example, an analysis of patients suspected to develop schizophrenia found a single dense network of highly interrelated symptoms and symptom subgroups across three different assessment scales [34]. A further analysis of patients with chronic schizophrenia and predominant negative symptoms computed from the Scale for the Assessment of Negative Symptoms (SANS) found preliminary evidence for a replicable negative symptom severity system as well as symptoms with high centrality, and showed that the baseline and endpoint symptom severity network consisted of symptoms including Affect, Poor responsiveness, Lack of interest, and Apathy-Inattentiveness. While the baseline and endpoint networks were similarly connected, both significantly differed from the change network where the Apathy-Inattentiveness symptom group split into three other groups [35]. Of note, in this study, the most central symptoms were Decreased Spontaneous Movements at baseline and endpoint, and Poverty of Speech for estimated change. Thus, this study found preliminary evidence for a replicable negative symptom severity system as well as for decreased Spontaneous Movements and Poverty of Speech having high centrality within those systems [35]. Treatment effects have also been informed by network analysis, with analysis of PANSS items showing that treatment-responsive patients had more densely connected symptom networks after antipsychotic treatment than at baseline, and symptom centralities increased following treatment [36]. Studies of this kind provide an idea of how network analyses could be applied to help provide future clinical practice insights and determine appropriate treatment targets for patients with schizophrenia.

The present article reports on a centrality network analysis that was performed on individual PANSS items in patient populations with acute exacerbation of schizophrenia or PNS. The aim of this study was threefold: (a) to evaluate connections between schizophrenia symptoms in two populations of patients (patients in acutely exacerbated phase of schizophrenia and patients with predominant negative symptoms [PNS]), and (b) to test whether network analyses support the Mohr 5 factor model of the PANSS and the Kahn 2 factor model of negative symptoms, and finally (c) to identify the most central symptoms in the two populations.

## Methods

### Participants

Two patient datasets were included in the analysis.

The first dataset consisted of patients from four phase II/III randomized, double-blind, placebo-controlled studies (*n* = 2193) investigating the efficacy of cariprazine in patients with an acute exacerbation of schizophrenia; detailed methods of the included studies have been published previously. Studies were similarly designed and included a 6-week double-blind treatment period (RGH-MD-03, RGH-MD-04, RGH-MD-05, and RGH-MD-16) [37–40]; the primary efficacy outcome in each study was mean change from baseline to week 6 in PANSS total score. This 30-item rating scale was specifically developed to assess both the positive and negative symptoms of schizophrenia, with each item scored on a 7-point scale and higher scores indicating greater severity. Inclusion criteria were a diagnosis of schizophrenia for ≥1 year and a current exacerbation <2 weeks in duration (<4 weeks in one trial [RGH-MD-03]), Clinical Global Impression-Severity (CGI-S) [41] baseline score ≥4 (moderately ill or worse), PANSS total score ≥80 and ≤120, and an item score ≥4 (moderate or higher) on ≥2 specified PANSS items (delusions, hallucinatory behavior, suspiciousness/persecution, and conceptual disorganization).

The second sample consisted of patients (*n* = 460) from the 26-week, randomized, double-blind trial with long-term (>2 year), stable schizophrenia and PNS; the primary efficacy measure was change from baseline to week 26 in PANSS factor score for negative symptoms (PANSS-FSNS). The PANSS-FSNS is based on the Mohr 5-factor model and consists of items N1 (blunted affect), N2 (emotional withdrawal), N3 (poor rapport), N4 (passive social withdrawal), N6 (lack of spontaneity and flow of conversation), G7 (motor retardation), and G16 (active social avoidance) [11]. Inclusion criteria were stable condition (i.e., no psychiatric hospital admissions, acute exacerbations, or imprisonments) for ≥6 months before screening; PNS for ≥6 months, with a PANSS factor score for negative symptoms (PANSS-FSNS) of ≥24, and a score of ≥4 on ≥2 of ≥3 core negative PANSS items at screening; <25% change from screening during a lead-in period [42]. Further, relevant positive (PANSS factor score for positive symptoms [PANSS-FSPS] ≥19 and >25% change from screening), depressive (Calgary Depression Rating Scale for Schizophrenia ≥6), and extrapyramidal symptoms (EPS; clinical judgment or Simpson-Angus Rating Scale score ≥3) were exclusionary. The PANSS-FSPS (based on the Mohr 5-factor model) is the sum of PANSS items P1 (delusions), P3 (hallucinations), P5 (grandiosity), P6 (suspiciousness/persecution), and G9 (unusual thought content) [11].

Cariprazine is a dopamine D_3_-preferring D_3_/D_2_ receptor partial agonist and serotonin 5-HT_1A_ receptor partial agonist approved for the treatment of adults with schizophrenia. The PANSS was administered across all these studies, which allowed for a unique opportunity to evaluate schizophrenia symptom connectivity for both the acute and PNS patient populations via network analysis. To maximize sample size, all patients with baseline values (even those without postbaseline data) from any treatment group (i.e., cariprazine, placebo, risperidone, and aripiprazole) were included in the performed network analyses in both datasets. Given this, only baseline PANSS data were used in the analyses.

### Statistical analysis

First, two network structures were estimated for all 30 items of the PANSS [43] in the two patient populations, as described above. From these network structures, data were analyzed in three ways: (a) to visualize the connections between PANSS items; (b) to investigate how well the network analysis supports the Mohr 5-factor model of schizophrenia and the Khan 2-factor model of negative symptoms; (c) to identify the most central symptoms in the two populations.

A network represents a system of nodes that are connected in one way or another [31, 32]. In network analysis, edges connect the different nodes, and its primary purpose is to identify the truly existing edges between the nodes. For this study, the nodes were the individual items of the PANSS, which were treated as ordinal variables, and the edges were the partial correlation coefficients between the different items. Therefore, the relationship between items is represented by an edge after controlling for all the other connections in a network. A weighted, undirected network was constructed, where the strength of the correlation between two items was represented by the thickness of a connecting line. For the network construction, the R package *qgraph* was used to compute the polychoric correlation matrix of the items. The graphical version of the least absolute shrinkage and selection operator (Glasso) algorithm, coupled with the extended Bayesian information criterion (EBIC) for model selection, was also applied as described previously [44].

We controlled for false positive edges by using the least absolute shrinkage and selection operator (lasso) method. The lasso method sets very small edges to zero, therefore pushing them out of the network estimation. To minimize the loss of true-existing edges, the default value of the tuning parameter of the Glasso algorithm was used to optimally balance between false positive and false negative edge identification rates. However, to study the potential influence of the tuning parameter on the network statistics, the network estimation was carried out after decreasing the false positive rate, with a concomitant increase in the false negative rate (e.g., increased loss of true relationships). Then, the networks constructed with no threshold for edge weights in Glasso (e.g., with even the smallest connections included; dense network) were also evaluated. The variation of the tuning parameter had little effect on the relative positions of the smallest and the strongest items in the network, as they remained below or above the average node strength value in most of the cases.

The importance of each item in the network was investigated using three measures: node strength (sum of all weighted connections), closeness (the multiplicative inverse of the sum of the length of the shortest paths between all other nodes and the specific node), and betweenness (number of times a node lies on the shortest path between two other nodes). All of these measures were automatically computed by *qgraph.* The results were graphically represented with nodes that have stronger and/or more connections between each other being placed closer together [43]. Afterward, node centrality was assessed based on node strength in both networks. Node centrality can be used to look at the structural importance of each node in a network [31, 32]. Node strength was chosen, as it stands for the direct influence of a node on the entire network.

Since the reliability of the obtained network is an important aspect in network estimation, the accuracy of the network statistics was investigated using the network simulation R package *bootnet* [44]. R codes used for the network estimation and to study network accuracy are shown in Supplementary Material. First, the accuracy of the edge weight and node strength estimations was evaluated by constructing nonparametric bootstrap confidence intervals (CIs). Significant differences (set at a level of 0.05) between nodes of different strengths were also studied by using the constructed CIs; the results were then visualized in a matrix format. In this study, the strongest and weakest nodes in the networks were compared; the validity of which was fully supported by the network accuracy tests. Finally, stability of the centrality order was investigated by sampling decreasing portions of the original population and expressing the correlation with the original result.

We did not prespecify any criteria to identify cluster groups (special communities) empirically in this data set. Instead, already existing, previously described clusters (i.e., the Mohr 5 factor and Khan 2 factor model) were investigated to examine how they behaved in this dataset of two different populations and which items would emerge as the most central items.

### The Mohr 5-factor model of PANSS and the Khan 2-factor model of negative symptoms

**Figure 1.**
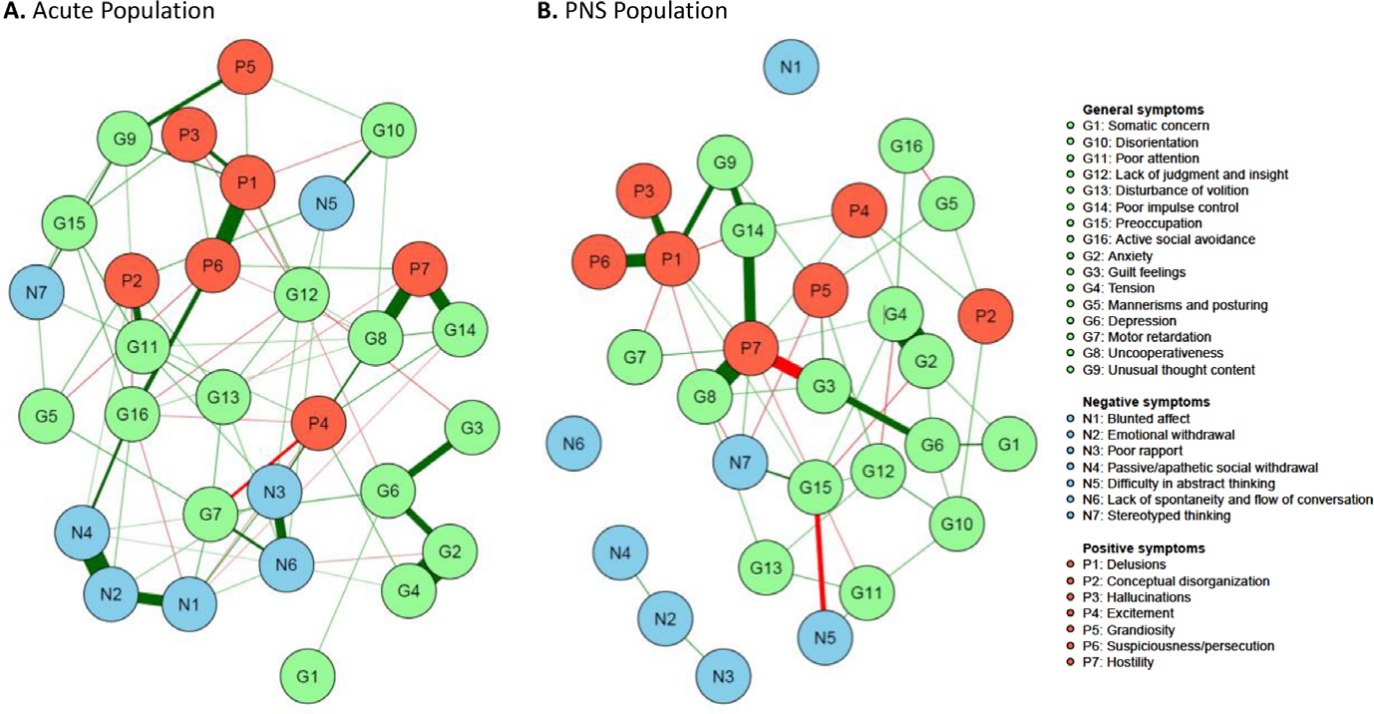
Network estimate of individual Positive and Negative Syndrome Scale (PANSS) items. (A) Acute population and (B) Predominant negative symptom (PNS) population. Nodes represent the different items with green, blue, and red symbolizing the items of the original PANSS general, negative, and positive symptoms subscale, respectively. The edges are shown by lines connecting the nodes with the width of the edge standing for the strength of the association. Green edges represent positive correlations, while red edges show negative ones. Nodes with more and stronger connections between each other are located closer to each other.

**Figure 2.**
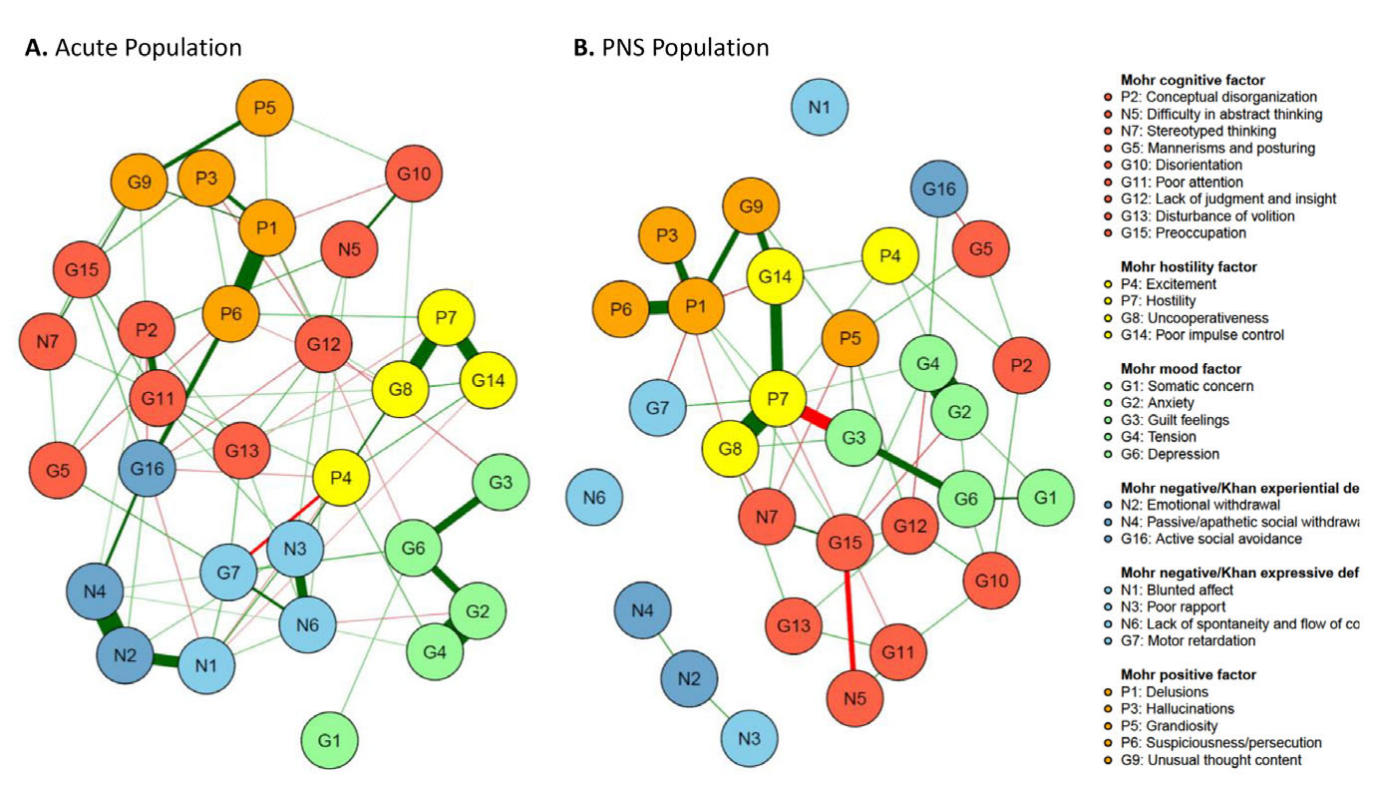
The Mohr 5-factor model and the Khan 2-factor model. (A) Mohr 5-factor and Khan 2-factor model in the acute population and (B) Mohr 5-factor and Khan 2-factor model in the predominant negative symptom (PNS) population. Nodes represent the different symptoms, and the edges are shown by lines connecting the nodes with the width of the edge standing for the strength of the association. Green edges represent positive correlations, while red edges show negative ones. Nodes with more and stronger connections between each other are located closer to each other.

For our analyses, we used the Mohr model to investigate the structure of all PANSS symptoms [11] and the 2-factor Khan model to analyze the structure of negative symptoms [18]. The five factors of the Mohr model are the cognitive, hostility, positive, negative, and mood factors; the 2 factors of the Khan model are the expressive and experiential factors. Corresponding items are listed in Figures 1 and 2. Using *qgraph*, we applied the Fruchterman–Reingold algorithm, which iteratively places nodes that are connected more strongly (strength and number of the connections) closer together to each other. The different factor models of the PANSS were then visually inspected in the network. In addition to visual inspection, we also applied a quantitative method to evaluate factor models by comparing the average connection strengths between PANSS items belonging to each specific factor versus the average of the other connections (e.g., connections between the factors). *p* values were based on a mixed model with Mohr factor components as levels of a fixed effect, and the edge weight as the independent variable.

## Results

### Study population

Baseline characteristics are summarized in Table 1.

**Table 1.** Patient demographics and baseline characteristics.

	Patients with an acute exacerbation	Patients with predominant negative symptoms
Age, mean (SD), years	37.8 (10.6)	40.5 (10.9)
Duration of illness, mean (SD), years	12.6 (9.8)	12.5 (8.7)
Male, *n* (%)	1552 (70.8)	264 (57.4)
PANSS total score, mean (SD)	96.4 (9.5)	76.6 (8.1)
PANSS-FSPS, mean (SD)	20.1 (3.3)	8.7 (2.7)
PANSS-FSNS, mean (SD)	23.0 (4.7)	27.6 (2.5)

*Note:* FSNS and FSPS were based on the Mohr 5-factor model [11].

Abbreviations: FSNS, Factor Score for Negative Symptoms; FSPS, Factor Score for Positive Symptoms; PANSS, Positive and Negative Syndrome Scale; SD, standard deviation.

### Network analysis of individual PANSS items

Figure 1 represents the network analysis of the PANSS items in patients with acute exacerbation of schizophrenia and in patients with PNS.

An important difference between the two patient groups was observed regarding the negative symptom items: in patients with an acute exacerbation, negative symptom items were connected to other symptoms (positive symptoms and general symptoms), while in patients with PNS, they were largely independent from one another and nearly isolated from all other symptoms. Moreover, considering connections within the negative symptom items, blunted affect (N1), emotional withdrawal (N2) and passive social withdrawal (N4), as well as poor rapport (N3) with lack of spontaneity (N6), formed two subgroups with strong within-group connections in the acute population (Figure 1A). In contrast, these items were nearly separated in the PNS population (Figure 1B).

In both populations, “difficulty in abstract thinking” (N5) and “stereotyped thinking” (N7) were far away from the other symptoms of the negative subscale.

### Applying the Mohr and Kahn factor models

The five Mohr factors, positive symptom factor = orange, hostility symptom factor = yellow, mood factor = green, cognition factor = red, and negative symptom factor = blue, which is split according to Khan’s proposal in experiential deficit (N2, N4, and G16 in darker blue) and expressive deficit (N1, N3, N6, and G7 in lighter blue), are shown in Figure 2. The visual representation illustrates how the symptoms of each of the five factors cluster rather well together in the acute population.

**Figure 3.**
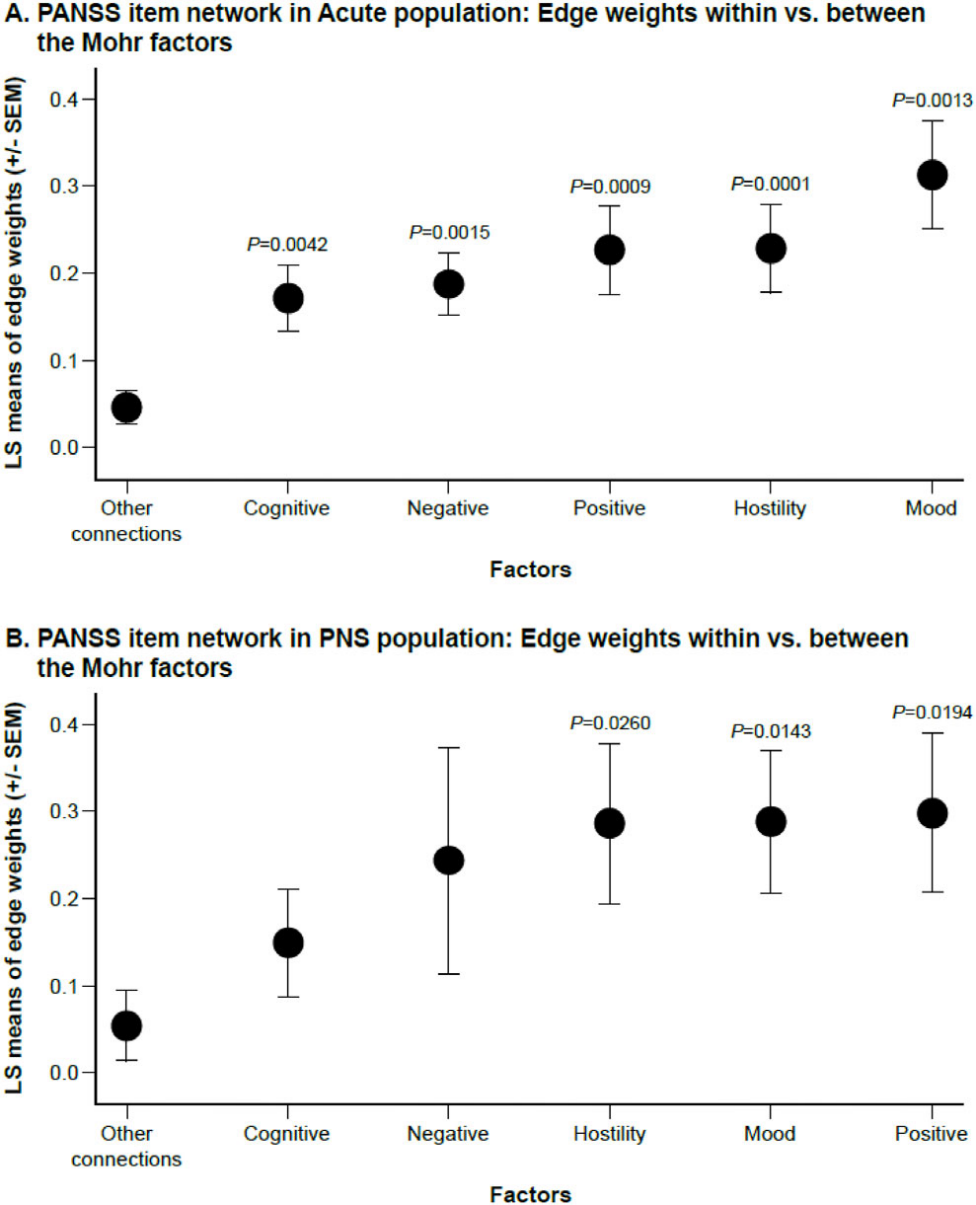
Connection strength for within-factor versus between-factor connections. (A) Acute population and (B) Predominant Negative Symptom (PNS) Population. *x*-axis represents least squares (LS) means of edge weights (±SEM) for each factor or for “other connections” (i.e., between factors). Comparisons are for average connection strength between items belonging to each specific factor versus the average of the rest of the connections.

Quantitative analysis of the item connectivity within factors confirmed findings from visual inspection, with higher average connection strengths within factors compared to the rest of the connections. Least square mean edge weights for within-factor connectivity reached statistical significance versus other connections for all five factors in the acute population (all, *p* < 0.01), and for the positive (*p* = 0.0194), hostility (*p* = 0.0260), and mood factors (*p* = 0.0143), but not the negative (*p* = 0.1719) or cognitive (*p* = 0.2025) factors (Figure 3).

The Khan model, which consists of experiential and expressive negative symptom factors, revealed a less clear-cut outcome. In the acute population, the experiential and expressive factors were visually recognized, although they did not distinctly separate. Blunted affect (N1), an expressive factor item, was strongly connected to emotional withdrawal (N2), an experiential factor item. Although the other expressive factor items (poor rapport [N3], lack of spontaneity and flow of conversation [N6], and motor retardation [G7]) clustered well together, we identified weak connections for motor retardation (G7) with experiential factor items N2 (emotional withdrawal) and N4 (passive social avoidance). Moreover, several items were linked with other, nonnegative factors too, such as G16 which was strongly connected to P6 (suspiciousness/persecution). In the PNS population, the expressive and experiential factors of the Khan model were not distinct, as N1, N2, N3, N4, and N6 rather stood as separate items, while G7 and G16 were more connected to the network. Motor retardation (G7) was positively linked to hostility (P7) and negatively linked to delusions (P1). Active social avoidance (G16) was positively connected to tension (G5) and negatively linked to mannerism and posturing (G5) (Figure 2).

### Centrality analysis

**Figure 4.**
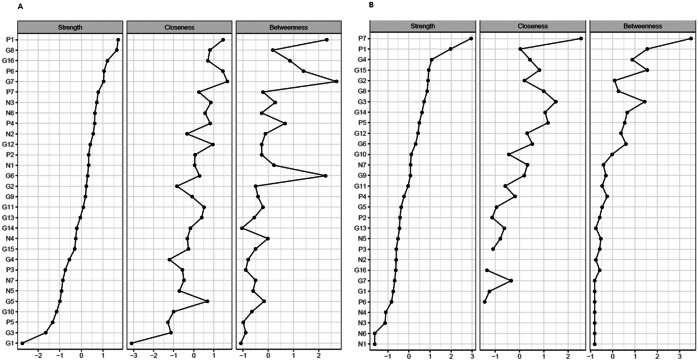
Node strength, closeness, and betweenness of individual PANSS items. (A) Acute population and (B) Predominant negative symptom population. The *x*-axis represents the normalized (*z*-score) values of the individual PANSS items for the three different network parameters. Individual PANSS items: P1, Delusions; P2, Conceptual disorganization; P3, Hallucinations; P4, Excitement; P5, Grandiosity; P6, Suspiciousness/persecution; P7, Hostility; N1, Blunted affect; N2, Emotional withdrawal; N3, Poor rapport; N4, Passive/apathetic social withdrawal; N5, Difficulty in abstract thinking; N6, Lack of spontaneity and flow of conversation; N7, Stereotyped thinking; G1, Somatic concern; G2, Anxiety; G3, Guilt feelings; G4, Tension; G5, Mannerisms and posturing; G6, Depression; G7, Motor retardation; G8, Uncooperativeness; G9, Unusual thought content; G10, Disorientation; G11, Poor attention; G12, Lack of judgment and insight; G13, Disturbance of volition; G14, Poor impulse control; G15, Preoccupation; and G16, Active social avoidance.

Further, node strength, closeness, and betweenness of individual PANSS items were also examined (Figure 4). In patients with acute exacerbation, the two strongest symptoms (e.g., those with the strongest node strength, which is determined by the number and strength of interactions) were delusions (P1) and uncooperativeness (G8), while in patients with PNS, they were delusions (P1) and hostility (P7). While for the patients in the acute studies, 5 out of the 7 Mohr negative symptoms were found in the 10 symptoms with the strongest node strength; in the PNS group, all 7 Mohr negative symptoms were found in the 10 symptoms with the lowest node strength. In addition, the results of the accuracy analysis (i.e., bootstrapped CIs for the edge weights, node strengths, difference matrix, and stability plot of the centrality order) are presented as Supplementary Figures S1–S4.

## Discussion

In this study, a network of individual PANSS items in a population of patients with an acute exacerbation of schizophrenia and in a population of patients with PNS was investigated.

While factor analyses investigate the interdependency of symptoms that belong to one cluster, network analyses can evaluate the relationship between clusters and single items as well [33]. An important finding of this study is that the negative symptoms were connected with other PANSS symptoms in the acute population, while the items of the negative symptom subscale became almost completely isolated in the PNS population. For example, in the acute population, many of the negative symptom items (i.e., blunted affect [N1], emotional withdrawal [N2], poor rapport [N3], passive social avoidance [N4], and lack of spontaneity [N6]) were strongly connected with one another and had connections with other symptoms as well. On the other hand, these negative items were nearly separated in the PNS population and had weak or no connections with each other. Although abstract thinking (N5) and stereotyped thinking (N7) separated from other PANSS negative subscale items in both populations, their connections differed. In the acute population, difficulty in abstract thinking (N5) and stereotyped thinking (N7) were linked to conceptual disorganization (P2), disorientation (G10) and an unusual thought content (G9). In the PNS group, difficulty in abstract thinking (N5) was negatively linked to preoccupation (G15), and stereotyped thinking (N7) was negatively related to uncooperativeness (G8). This may support the notion that difficulty in abstract thinking (N5) and stereotyped thinking (N7), originally thought to be negative symptoms, rather contribute to the cognitive symptom factor [13]. Moreover, findings are in line with a recent consensus paper of the European Psychiatric Association mentioning that if first generation scales such as the PANSS are used for negative symptom studies, then only items N1, N2, N3, N4, and N6 should be considered for negative symptom analyses as the rest is describing other symptoms [30].

This study also investigated whether this network analysis can support the Mohr 5-factor model of PANSS symptoms [11]. The Mohr 5-factor PANSS model consists of symptom factors representing cognitive, mood, negative, positive, and hostility symptoms. In this network analysis, the 5-factor model was best recognized in the acute population and less so in the PNS population. Quantitative analysis of item connectivity within each of the five Mohr factors confirmed these visual observations, indicating significantly greater within-factor connectivity compared with other connections for all five factors in the acute population. In the PNS population, within-factor connectivity was significantly stronger than between-factor connectivity for the positive, hostility, and mood factors, but not for the negative or cognitive factors. For example, in patients with PNS, mannerism and posturing (G5) and conceptual disorganization (P2), which are items included in the Mohr cognitive factor, are separated from the other cognitive items. The same applies to blunted affect (N1) and active social avoidance (G16), which sit very far from other items of the negative symptoms factor in patients with PNS. Although disorientation (G10), lack of judgment (G12), and difficulty in abstract thinking (N5) items have relations with other noncognitive items and visually look separated from cognitive items in the acute population, the within-factor connections were significantly stronger than the between-factor connections.

With regard to the negative symptom constructs, exploratory and confirmatory factor analyses suggested a 2-factor structure across a variety of scales [17, 18, 45, 46]. The Khan 2-factor model that was explored in this article, is characterized by an experiential factor (items N2, N4, and G16) and an expressive factor (N1, N3, N6, and G7). In our network analysis, these two negative symptom factors were well delineated only in the acute population, but even there, connections were identified between the two Kahn factors: emotional withdrawal (N2) was strongly connected to blunted affect (N1), and moderately linked to motor retardation (G7), while passive social withdrawal (N4) had a moderate connection with motor retardation (G7) as well as with lack of spontaneity and flow of communication (N6). As mentioned earlier, negative factor items in acute patients showed between-factor connections as well: One of them is a strong link between active social avoidance (G16) and suspiciousness/persecution (P6), suggesting that active social avoidance may be a consequence of paranoia and represents a secondary negative symptom. The structure of negative factor items in the PNS population suggests that blunted affect (N1), emotional withdrawal (N2), poor rapport (N3), passive social withdrawal (N4), as well as lack of spontaneity and flow of communication (N6) are separate dimensions of primary negative symptoms. In contrast, motor retardation (G7; a reduction in motion activity with slowing or lessening of movements and speech) and active social avoidance (G16; diminished social involvement with unwarranted fear hostility or distrust) may be consequences of other symptoms and represent predominantly secondary negative symptoms. However, this suggestion requires further study. Despite being mainly separate entities, emotional withdrawal (N2) was moderately connected to poor rapport (N3) and passive social avoidance (N4) in the PNS group. Although the two populations showed different latent structures of the negative symptom construct, emotional withdrawal (N2) seemed to have a relatively well-connected position among negative symptoms in both groups.

These findings suggest that primary negative symptoms are better conceptualized as five distinct negative symptom dimensions on the PANSS, and complement the results of the CAINS and BNSS studies, where a 5-factor model of negative symptoms was reported to be a better fit than 2-factor models, measured by both factor and network analyses [20, 33, 47]. According to our results, the 2-factor model could be still applicable for secondary or mixed negative symptoms.

Results of network analysis were further analyzed to calculate centrality statistics. Based on node strength, delusions (P1) and uncooperativeness (G8) were the two centers in patients with an acute exacerbation, while delusions (P1) and hostility (P7) were the two centers of symptoms in patients with PNS. These central items belong to identical Mohr factors in both populations: the positive symptom factor (P1) and hostility/excitement factor (G8 and P7). Delusions, uncooperativeness, and hostility have high clinical relevance in terms of the ability to build a therapeutic relationship with these patients. Based on closeness and betweenness evaluations, slightly different conclusions about the central symptoms could be made. However, the trend for connections in the centrality analyses between negative symptoms was consistent based on all three measures (node strength, closeness, and betweenness), making it clear that negative symptoms have loose connections in the PNS population.

Taken together, our findings suggest that the same negative symptom items are measuring separate dimensions in the two patient populations. Our interpretation of this result is that the observed network of the negative symptoms may represent their secondary versus primary nature in the acute versus PNS populations, respectively [27]. While secondary negative symptoms usually improve when positive symptoms are effectively managed by dopamine D_2_ receptor antagonists or partial agonists [27], primary negative symptoms generally do not respond to these antipsychotics, and their management may require different mechanisms (e.g., agents that target D_3_ receptors) [42, 48–50]. Hence, better differentiation between primary and secondary negative symptoms of schizophrenia could have a high impact on day-to-day therapy and related outcomes.

Several limitations need to be considered when interpreting the results of this study. First, only studies involving cariprazine were included; however, since analyses involved only baseline ratings, the investigated drug in the randomized trials should not affect results significantly. Nevertheless, similar analyses from future studies should be conducted, as inclusion criteria can vary across trial programs. In addition, the connections between items should be interpreted with caution in the PNS population because patients with positive symptoms were excluded and no definitive causal statements can be made as the data was cross-sectional in nature. Second, patient numbers were different; for acute population, four studies (*n* = 2193) were available, while only one study (*n* = 460) contributed to the PNS population. A smaller sample size can affect the accuracy of the network estimation, as larger sample sizes provide more accurate estimations. This fact is reflected by the different simulated CI pattern and network stability between the two networks. However, since the relative order of the items within each population was compared between the two populations, only the strongest and weakest connections were emphasized. Third, although rater training was conducted, the precision and interrater reliability of the PANSS ratings is always a limitation in such studies. Fourth, not all five domains of negative symptoms could be evaluated due to a limitation of the PANSS, which does not include anhedonia, a key negative symptom [29]. Our network analyses are based on the Mohr factor model [11]. Clearly, several other schizophrenia symptom factor structures, with different levels of empirical support, have been proposed, including the factors proposed by Lindenmayer [9], Marder [10], and Wallwork [12]. While comparing network analysis results across the different proposed factor models would be a potentially fruitful endeavor, we chose the Mohr model a priori. Future research might want to compare network analytic results based on the choice of symptom factor structure to advance methodological research in this area. Fifth, the current analyses focused on endpoint scores. Although a network analysis of change scores would also be of interest, applying this additional approach was outside of the scope of the current manuscript, but should be addressed in future analyses. Lastly, baseline values for depressive symptoms and EPS, which are symptoms that can confound negative symptom outcomes, were not characterized in the acute study population and were therefore not evaluated in this analysis. Regardless of these limitations, results from these analyses are informative regarding the PANSS factor structure, especially in relation to differences in the negative factor in acutely exacerbated and in PNS patients with schizophrenia.

In conclusion, the main finding in this network analysis is that while negative symptoms in patients with an acute exacerbation of schizophrenia were connected with the other PANSS symptoms and considered mainly secondary, in the PNS population they were neither connected to other symptoms, nor to each other and therefore they were considered mainly primary, separate symptom dimensions. This structure provides general support for a 5-factor model for primary negative symptoms. It is hoped that these results can help further subdivide patients with schizophrenia in regard to illness trajectory, treatment choice, and treatment outcome.

## Data Availability

The data generated for this article are available from the corresponding author on reasonable request. All shared data must be used for noncommercial purposes.

## Supplementary Materials

To view supplementary material for this article, please visit http://doi.org/10.1192/j.eurpsy.2021.2241. click here to view supplementary material
