# Supplementary material for: Disentangling the symptoms of schizophrenia: Network analysis in acute phase patients and in patients with predominant negative symptoms
Source: Eur Psychiatry. 2021 Oct 13;65(1):e18. doi: 10.1192/j.eurpsy.2021.2241 (PMC8926909; doi:10.1192/j.eurpsy.2021.2241)
Supplement: Supplementary file 1 [file S0924933821022410sup001.zip › S0924933821022410sup001.pdf]

Supplemental Figure 1. Bootstrap Confidence Intervals for Edge Weights

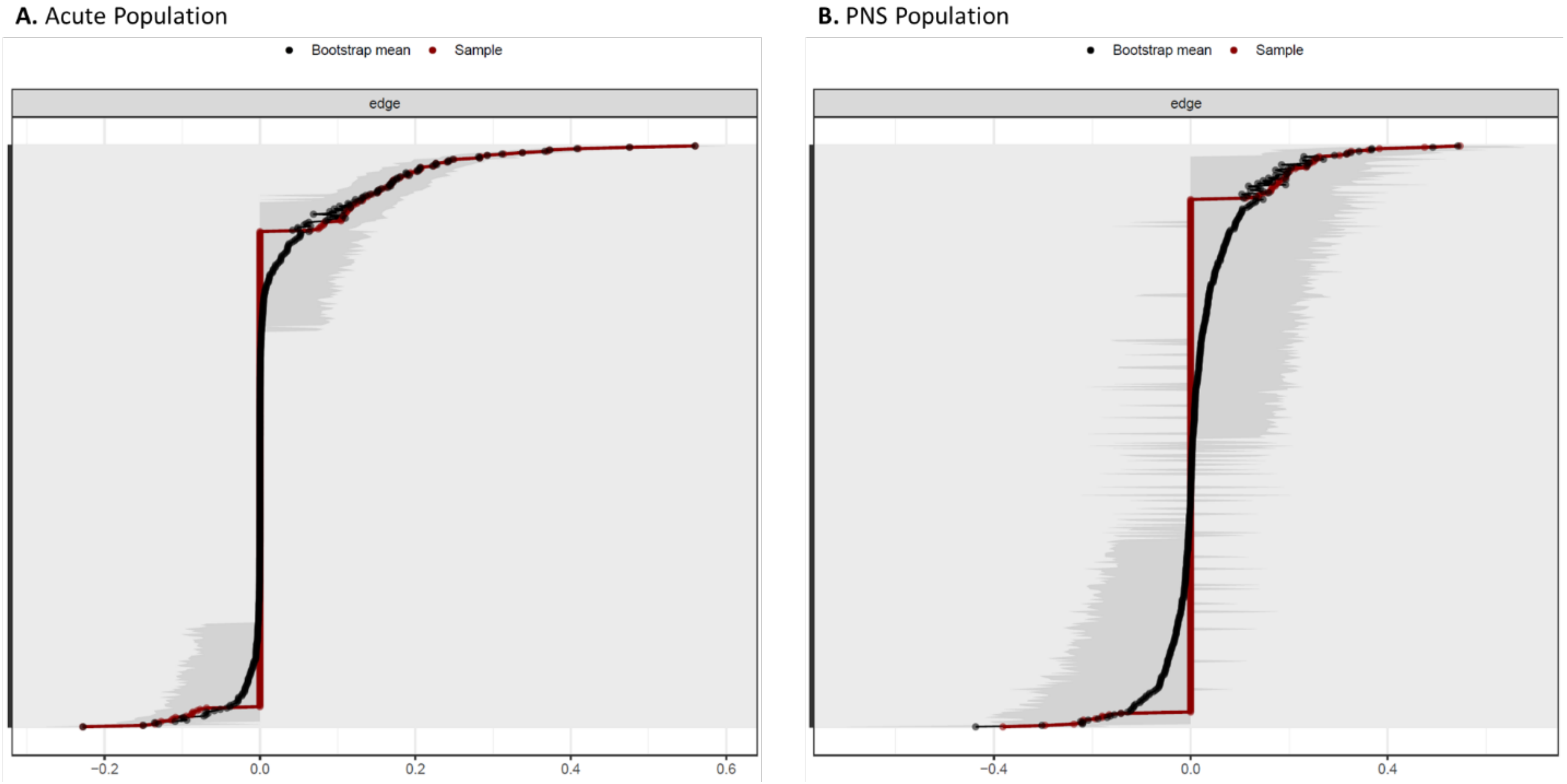

(A) Acute population, (B) Predominant negative symptom (PNS) population.
In both patient populations, there is overlap between the sample and bootstrap means. Due to the high density of the item pairs, only the range of the edge weight values are shown without the names of the individual pairs. Sample edge weights equal to zero indicate weak relationships omitted by the network estimate procedure.
