# Supplementary material for: Disentangling the symptoms of schizophrenia: Network analysis in acute phase patients and in patients with predominant negative symptoms
Source: Eur Psychiatry. 2021 Oct 13;65(1):e18. doi: 10.1192/j.eurpsy.2021.2241 (PMC8926909; doi:10.1192/j.eurpsy.2021.2241)
Supplement: Supplementary file 1 [file S0924933821022410sup001.zip › S0924933821022410sup002.pdf]

Supplemental Figure 2. Bootstrap Confidence Intervals for Node Strengths

A. Acute Population

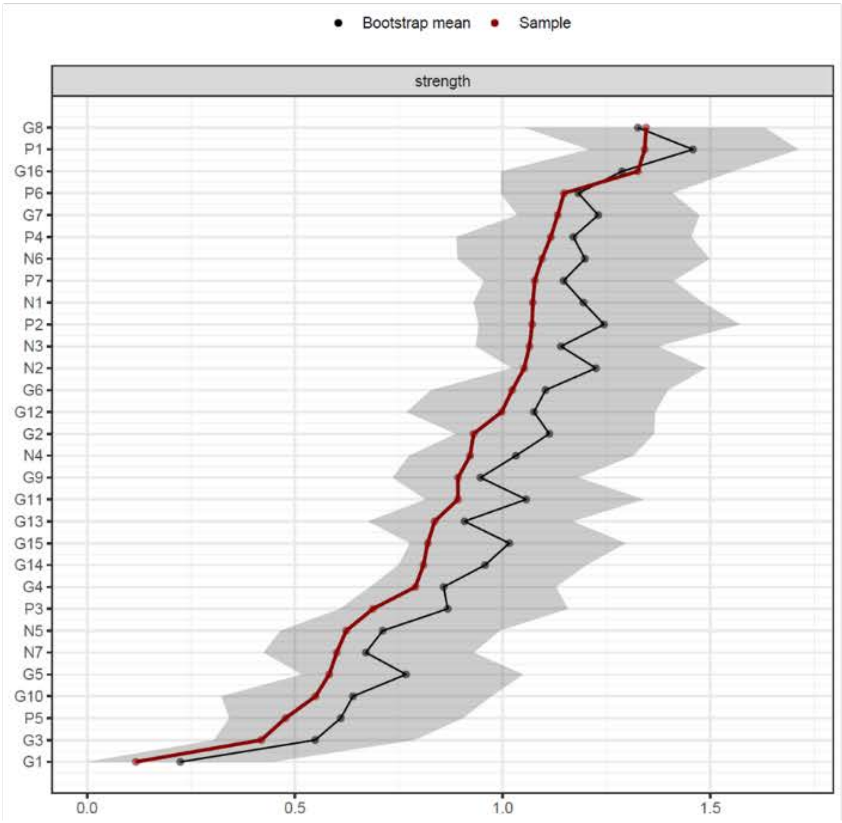

B. PNS Population

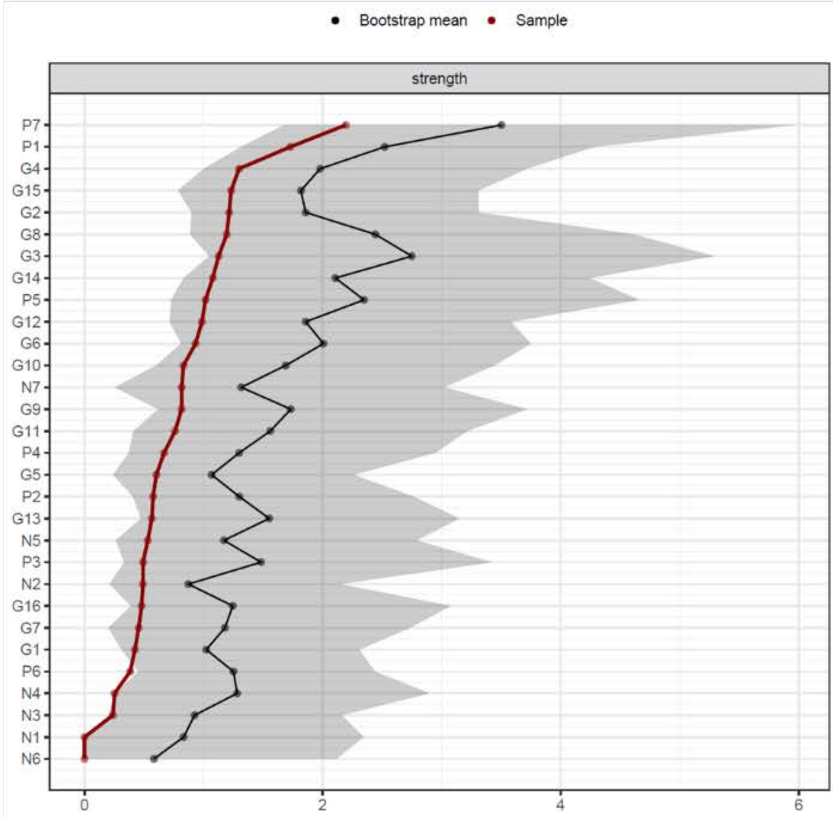

(A) Acute population, (B) Predominant negative symptom (PNS) population.

The majority of the confidence intervals for the sample and bootstrap means overlap with each other, especially in PNS population, indicating that statistically significant differences can be expected between the nodes with the highest and lowest values. Note that the CIs in the PNS population are wider compared to the acute population, which may be due to its smaller sample size; however, the tendency of the nodes to occupy the highest or weakest strength position are well reflected by the simulation.
