# Supplementary material for: Disentangling the symptoms of schizophrenia: Network analysis in acute phase patients and in patients with predominant negative symptoms
Source: Eur Psychiatry. 2021 Oct 13;65(1):e18. doi: 10.1192/j.eurpsy.2021.2241 (PMC8926909; doi:10.1192/j.eurpsy.2021.2241)
Supplement: Supplementary file 1 [file S0924933821022410sup001.zip › S0924933821022410sup003.pdf]

Supplemental Figure 3. Difference Matrix of the Nodes

A. Acute Population

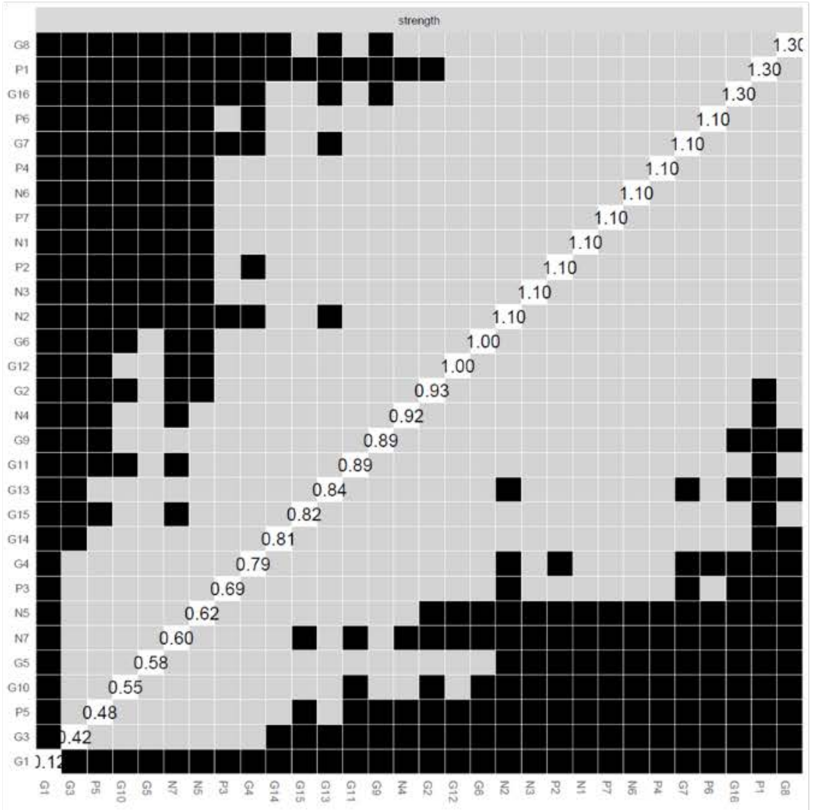

B. PNS Population

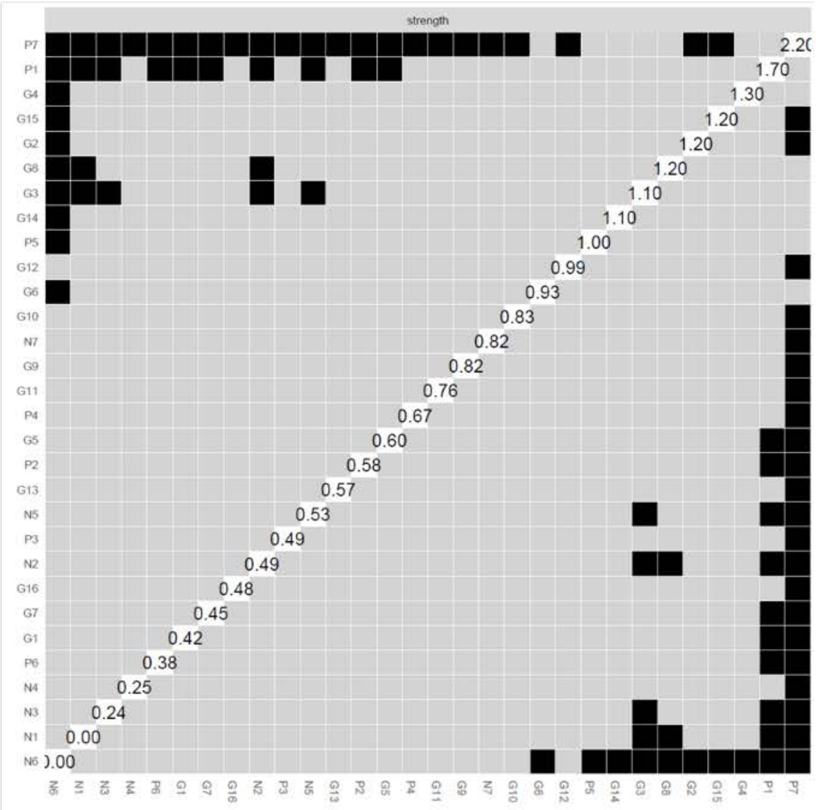

(A) Acute population, (B) Predominant negative symptom (PNS) population.
Black boxes indicate statistically significant differences between pairs of nodes regarding the node strength values, suggesting the validity of the conclusions are related to the importance of the strongest and weakest items within the populations.
