# Supplementary material for: Disentangling the symptoms of schizophrenia: Network analysis in acute phase patients and in patients with predominant negative symptoms
Source: Eur Psychiatry. 2021 Oct 13;65(1):e18. doi: 10.1192/j.eurpsy.2021.2241 (PMC8926909; doi:10.1192/j.eurpsy.2021.2241)
Supplement: Supplementary file 1 [file S0924933821022410sup001.zip › S0924933821022410sup004.pdf]

Supplemental Figure 4. Stability Plot of the Centrality Order

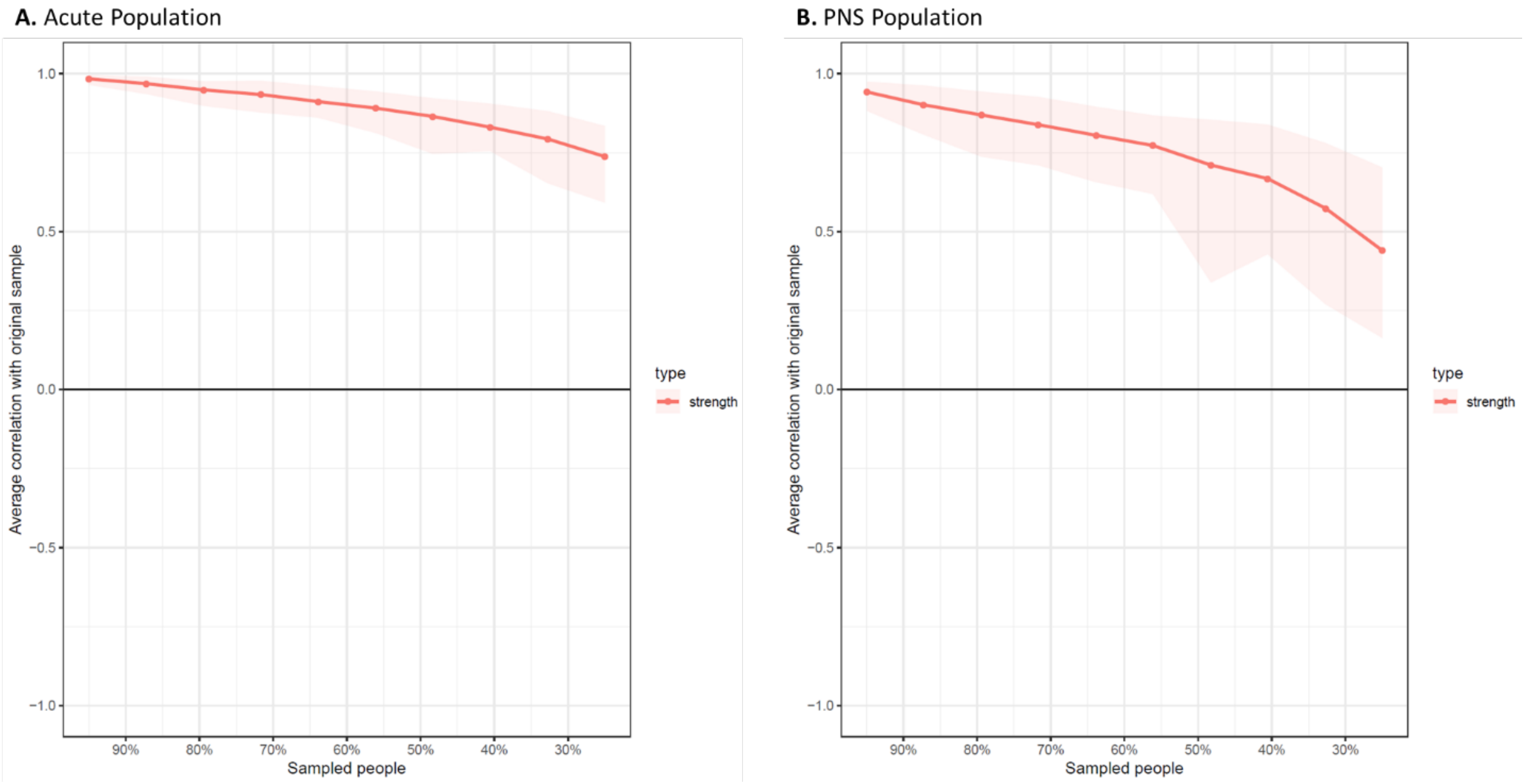

(A) Acute population, (B) Predominant negative symptom (PNS) population.

Decreasing portions of the patient population were sampled, and the average correlations (with 95% confidence interval) are presented. Note that the network for the PNS population was less stable, which may be due to its smaller sample size; however, after reducing the sample size to 50%, the average correlation with the results of the full population is preserved at a level of at least 0.75. This is an indication of the robustness of the network estimation.
