# Supplementary material for: Disentangling the symptoms of schizophrenia: Network analysis in acute phase patients and in patients with predominant negative symptoms
Source: Eur Psychiatry. 2021 Oct 13;65(1):e18. doi: 10.1192/j.eurpsy.2021.2241 (PMC8926909; doi:10.1192/j.eurpsy.2021.2241)
Supplement: Supplementary file 1 [file S0924933821022410sup001.zip › S0924933821022410sup005.pdf]

Supplementary material: R code

```
library("qgraph")
library("psych")
library("bootnet")
rm(list=ls(all=TRUE)) #remove all objects

# read in data
indata_0 <- read.table("C:/Users/acsaik/acute.txt", header=TRUE, sep=",")
head(indata_0 )

# PANSS items
names <- scan("Minta.txt", what = "character", sep = "\n")
itemgroups <- c(rep("General symptoms",16), rep("Negative symptoms",7),
rep("Positive symptoms",7) )

        indata_1 <- subset(indata_0) # for the case any subset is needed
        indata_2 <- indata_1[c(-1,-2,-3)] #   only the items are needed
        head(indata_2 )

#####################################################################################
#
#
# method used in the paper: network structure with QGRAPH, graphical lasso (glasso):
estimates this, where edges are the partial corr coeffs
# control false+ edges using Least Absolute Shrinkage and Selection Operator (lasso)
# choose the  shrinkage parameter to minimize extended BIC (EBIC)
#
#####################################################################################
#

# correlation matrix
corMat <- cor_auto(indata_2)
write.table(corMat , "1b_corMat.csv", row.names=T, col.names=T)

#####################################################################################
#
#
# Network structure and edge strength ...
#
#####################################################################################
#

tuning <-0.008
simN <- 1000
FalseOrNot <- TRUE
pdf("AcutePatients_Panss_tuning_0.008.pdf")


# NETWORK
        Graph_pcor <- qgraph(corMat,
                graph = "glasso",
```

```
                layout = "spring",
                lambda.min.ratio = tuning,
                threshold= FalseOrNot , #TRUE,
                sampleSize = nrow(indata_2),
                nodeNames = names,
                groups = itemgroups,
                color = c("PaleGreen", "SkyBlue", "Tomato" ),
                edge.width = 2,
                #title = "Network structure",
                legend.cex= 0.25)


# CENTRALITY
        centRes <- centrality(Graph_pcor)

        centralityPlot(Graph_pcor,
                scale="z-scores",
                orderBy="Strength",
                decreasing=FALSE,
                include=c("Strength","Closeness","Betweenness"))
        centralityPlot(Graph_pcor,
                scale="raw",
                orderBy="Strength",
                decreasing=FALSE,
                include=c("Strength","Closeness","Betweenness"))


# Node strength (degree):
        centRes$OutDegree # or InDegree, it is the same in unweighted networks

# Betweenness
        centRes$Betweenness




##################################################################################
#
##################################################################################
#
#
# Bootnet package network estimation and bootstrap, to produce CI, stability
#
##################################################################################
#

# this should produce the same network
        Network <- estimateNetwork(indata_2,
                        default = "EBICglasso",
                        lambda.min.ratio = tuning ,
```

```
                        threshold= FalseOrNot ,
                        )


        plot(Network, layout = 'spring')
        centralityPlot(Network, scale="z-scores", orderBy="Strength",
decreasing=FALSE, include=c("Strength","Closeness","Betweenness"))


####################################################################################
###
####################################################################################
###
#
# Bootstrap
#
####################################################################################
#####

#######################################
#
# Accuracy of edge weight, node strength, with conf int
#
#######################################

        Results1 <- bootnet(Network, nBoots = simN, nCores = 8, default =
"EBICglasso", type = "nonparametric")
                # type =
c("nonparametric","parametric","node","person","jackknife","case")


        plot(Results1, statistics = "edge",
                labels = FALSE, order = "sample")

        plot(Results1, statistics = "strength",
                plot = "area",
                CIstyle = "quantiles",
                sampleColor ="darkred",
                samplelwd = 1,
                meanColor = "black",
                meanlwd= 0.5,
                bootColor = "black",
                bootlwd= 0.9,
                areaAlpha = 0.2,
                order = "sample",
                decreasing = TRUE)

        plot(Results1, statistics = "Strength",
                plot = "difference",
                order = "sample",
```

```
                decreasing = TRUE )


#################################
#
# Centrality stability
#
#################################


        Results2 <- bootnet(Network, nBoots = simN, nCores = 8,type = "case")


# Compute CS-coefficients:

        corStability(Results2, cor = 0.7, statistics = "strength", verbose = TRUE)

        plot(Results2,
        statistics = "strength")


dev.off()
```
